# Right Middle Cerebral Artery Occlusion Following Ovarian Hyperstimulation Syndrome: A Case Study

**DOI:** 10.7759/cureus.99808

**Published:** 2025-12-22

**Authors:** Yi Hui Liu

**Affiliations:** 1 Obstetrics and Gynaecology, St George Hospital, Sydney, AUS

**Keywords:** arterial thromboembolism event, case study, gynecology, obstetrics, ovarian hyperstimulation syndrome

## Abstract

Ovarian hyperstimulation syndrome (OHSS) is one of the most serious complications occurring from the growing field of artificial reproductive technology. The expanding literature points to a link between OHSS and thromboembolism events. We present a case of ischemic stroke secondary to a right middle cerebral artery occlusion in a female after a fresh embryo transplant. A CT of the abdomen demonstrated significant free fluid in the abdomen, in which hemoperitoneum from an ectopic pregnancy could not be excluded. A paracentesis was completed and demonstrated clear fluid, thus supporting a presumed diagnosis of OHSS. The patient was able to get urgent endovascular clot retrieval and continues to have a progressive pregnancy at the time of publication.

## Introduction

Ovarian hyperstimulation syndrome (OHSS) is a rare but severe complication of assisted reproductive technology (ART). While the reported prevalence of severe forms of OHSS ranges from 0.5% to 5%, there is a lack of evidence-based data for predictive factors of OHSS, thus making it difficult to predict and prevent [1,2]. Clinical manifestation varies in severity from nausea and vomiting to ascites, pleural effusion, dyspnea, and thromboembolism events [3]. The pathophysiology is hypothesized to be secondary to the production of multiple angiogenic and vasoactive cytokines by the multiple corpora lutea, which causes increased vascular permeability and massive fluid shift from the intravascular compartments into third spaces [4]. This fluid shift can potentially lead to decreased perfusion to organs, increased hemoconcentration, and an increased risk of thromboembolic events [5,6].

As the use of ART continues to rise worldwide, there has been an increase in cases of OHSS and, subsequently, an increased risk of complications, including thrombotic events. While most thromboses that occur secondary to OHSS are primarily venous, those that are arterial are primarily intracerebral, thus carrying risks for higher morbidity and mortality [7,8]. Here, we illustrate a case of right middle cerebral artery (MCA) occlusion secondary to OHSS.

## Case presentation

**Figure 1:**
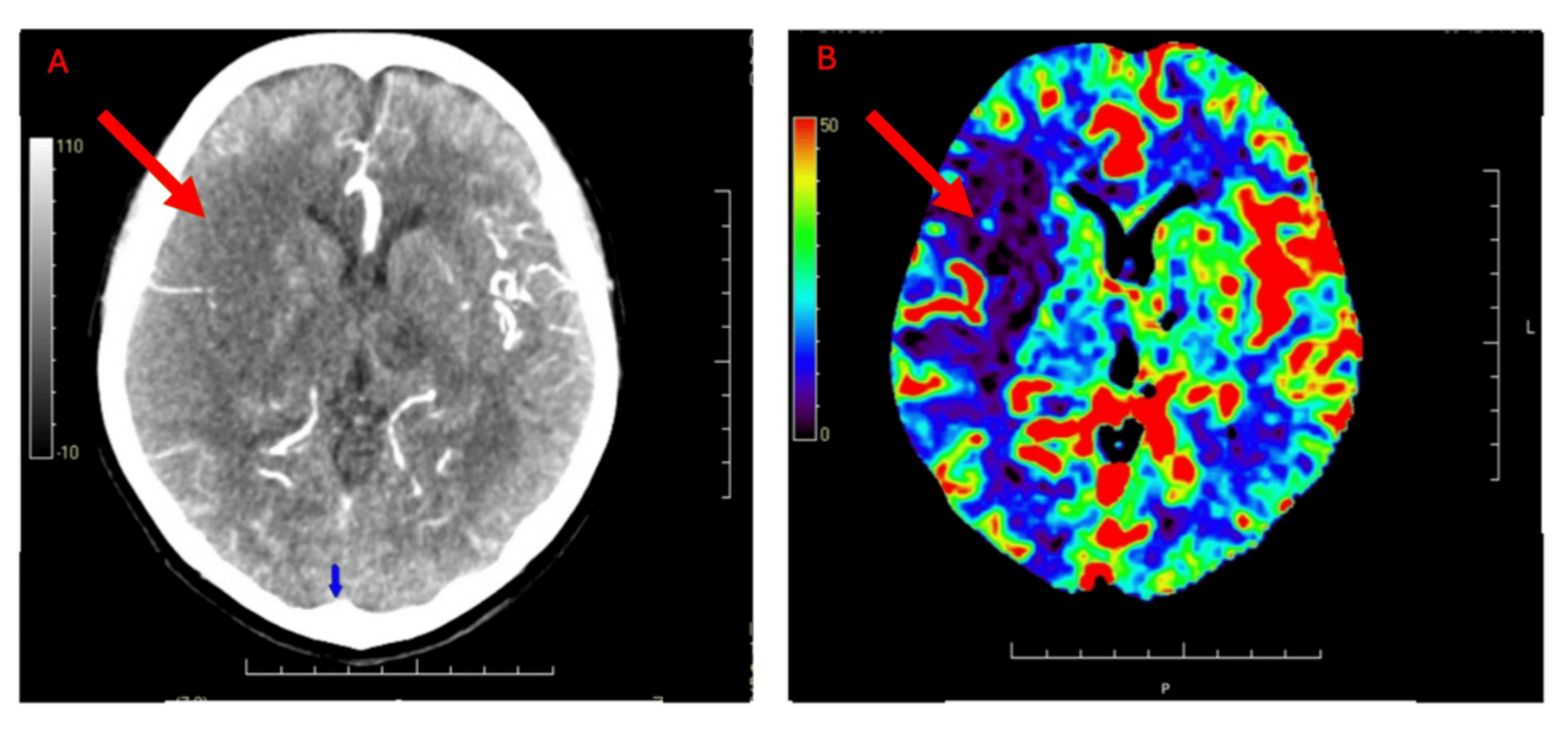
CT stroke study: cerebral CT and perfusion imaging. (A) A non-contrast CT scan of the head shows an area of cortical hypoattenuation and loss of gray–white differentiation in the right frontal region (red arrow), suspicious for acute ischemia. (B) CT perfusion map demonstrates reduced cerebral blood flow (red arrow) in the corresponding right frontal region, consistent with acute hypoperfusion.

**Figure 2:**
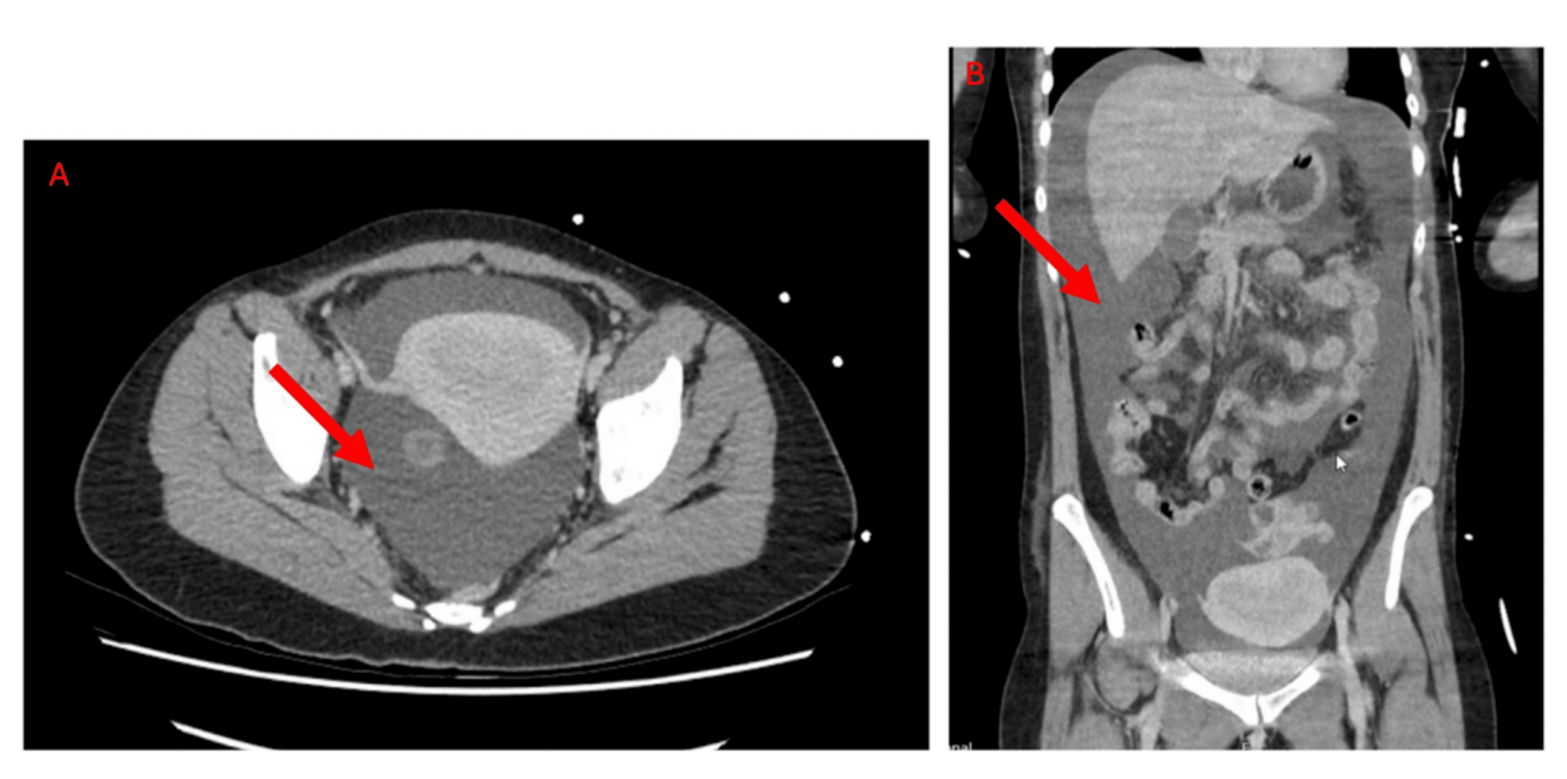
CT of the abdomen and pelvis. (A) Axial contrast-enhanced CT showing free fluid present in the pelvis (red arrow). (B) Coronal CT demonstrating more extensive intra-abdominal free fluid tracking superiorly (red arrow).

A 39-year-old nulliparous woman was brought in by ambulance for left-sided dense paresis with bilateral ocular deviation to the right. She was seen as a Category 1 triage case in the emergency department. Initially, the patient was hypotensive in the ambulance but was stabilized with intravenous fluid resuscitation with crystalloid. Throughout her review in the emergency department, she was hemodynamically stable with a hemoglobin of 151 g/L, normotensive, and normocardia. She had a Glasgow Coma Scale score of 13 and a National Institutes of Health Stroke Scale score of 17. Her abdomen was distended but non-tender with nil abdominal guarding or rebound tenderness. A collateral history confirmed an ART pregnancy for secondary fertility of unknown cause with fresh embryo implantation nine days prior. She had no other past medical history, no regular medications, and no known family history of thrombophilia. Her biochemistry demonstrated mildly low albumin (26 g/L) but a normal hematocrit (0.406 L/L). A focused assessment with sonography in trauma (FAST) scan was completed, showing four quadrants of free fluid. An urgent CT stroke study and CT abdomen/pelvis was completed, demonstrating dense right M1 occlusion with a mismatch of the right MCA territory and preserved cerebral blood volume (Figure 1), and a significant amount of peritoneal free fluid, which could not exclude hemoperitoneum with the Hounsfield unity density reading (Figure 2). A bedside paracentesis was performed, showing clear, serous fluid with nil blood staining, thus supporting a presumed diagnosis of OHSS rather than a ruptured ectopic pregnancy.

The impression was an acute R1 M1 stroke in the context of OHSS and pregnancy, and the patient was a candidate for hyperacute intervention. While there were initial discussions of whether a pelvic ultrasound was needed to exclude an ectopic pregnancy, the multidisciplinary team had decided that, first, the timeframe of this early pregnancy would make it difficult for ultrasound to accurately confirm an intrauterine pregnancy, second, the serous, non-blood tinged fluid of the paracentesis would be unlikely in keeping with a ruptured ectopic pregnancy, and, lastly, the time-sensitive nature of a stroke necessitated immediate management. In conjunction with the neurology team, a decision was made to transfer the patient to another tertiary center for endovascular clot retrieval, which was successful. She was then transferred back for inpatient rehabilitation. While inpatient, her beta-human chorionic gonadotropin level had risen appropriately, and her hematocrit had continued to fall, with the lowest four days after her initial presentation at 0.349 L/L. An ultrasound was completed, demonstrating a live baby with normal growth parameters. She is currently attending routine outpatient antenatal care and rehabilitation to regain function in her affected side.

## Discussion

This case adds to the growing literature of thromboembolism following OHSS and demonstrates important features for clinicians to be aware of when making a presumed diagnosis of OHSS. The current theory for the pathophysiology of OHSS is due to an excess production of angiogenic and vasoactive cytokines from the multiple corpora lutea, thus allowing for increased vascular permeability and third spacing, causing sequelae such as ascites, hydrothorax, and hydropericardium. In response to the fluid shift, there is an increase in hemoconcentration and hypovolemia with resultant arterial hypotension [5,6]. The resultant arterial hypotension decreases the blood flow velocity while the increase in estradiol concentration during ovulation induction increases the risks of clotting, thus overall increasing the risks of thrombi formation [9]. Given the timing of ovulation induction with the timeframe needed for this cascading effect, more cases of thromboembolic phenomenon have been reported to occur in late stages of OHSS, which is defined as 12-17 days after ovulation or ovulatory trigger [3,10]. These were all illustrated in our case, which demonstrated a patient who was nine days after a fresh embryo transfer and 14 days after an ovulation trigger with clinical signs, including abdominal distension. Her mildly low albumin and falling hematocrit were suggestive of third spacing in keeping with the pathophysiology of OHSS.

While this case demonstrates signs and symptoms for clinicians to recognize when making a presumed diagnosis of OHSS, it also illustrates two unique considerations. The first is the use of paracentesis for immediate exclusion of a ruptured ectopic pregnancy. At the time of the patient’s initial assessment, she had a positive urine pregnancy test but had not yet had an intrauterine pregnancy confirmed on ultrasound. A FAST scan was completed, demonstrating four quadrants of free fluid. The CT of the abdomen was only able to visualize a bulky uterus, given that the views were obscured by extensive fluid (Figure 2). As the patient was for consideration of thrombolysis and a pelvic ultrasound was unlikely to accurately demonstrate an intrauterine pregnancy this early on, further investigations were needed to presume a diagnosis. Clinically, the patient did not demonstrate signs of an ectopic pregnancy, including guarding or rebound tenderness [11]. Through a bedside paracentesis, we were able to provide symptomatic relief for the patient while simultaneously assessing the fluid for any hemoperitoneum. The fluid was clear and allowed us to proceed with bleeding risks reasonably mitigated for the impending procedure.

Another discussion point for this case would be the growing association between OHSS and arterial thromboembolism. As ART continues to grow in practice, clinicians should be aware of the newly developing risk for intravascular thromboembolism. Current research has not been able to demonstrate strong evidence between the incidence of OHSS and pretreatment characteristics, including age, body mass index, allergies, blood group, and etiology of sterility [2]. Further studies will need to be completed to delineate risk factors for patients developing OHSS and the sequelae of thromboembolism events. Fertility specialists should also consider screening patients for any predisposing factors and initiating patients on thromboprophylaxis to help mitigate any risks.

## Conclusions

As the use of ART continues to expand worldwide, the incidence of OHSS will continue to rise. This case demonstrates the use of paracentesis to help delineate between OHSS and an ectopic pregnancy in an emergent situation, as well as the need for further research to evaluate individual risk factors to consider the commencement of thromboprophylaxis in high-risk patients.
